# Iodoform in Dental Surgery

**Published:** 1884-03

**Authors:** C. F. W. Bodecker

**Affiliations:** New York


### IODOFORM IN DENTAL SURGERY.

BY C. F. W. BODECKER, D. D. S., M. D. S., NEW YORK.

Iodoform was discovered in 1822 by Serullas; it is obtained by the action of iodine upon alcohol, in the presence of an alkali; it forms into small, scale-like crystals, of a light, yellow lemon color, of a very disagreeable odor, and peculiar sweetish taste. Iodoform is insoluble in water, but freely soluble in ether, chloroform, and in fats and oils. When exposed to the atmosphere it gradually evaporates, even at an ordinary temperature. In solution it gradually decomposes, whereby free iodine is liberated ; especially is this the case when exposed to the sun or daylight. All solutions of iodoform should therefore be kept in a dark and cool place, but even with the greatest of care they decompose in from one to two weeks, when the solution assumes a dark, yellowish brown color. To disguise the very disagreeable odor several substances have been mentioned, such as the oils of peppermint and wintergreen, or cumarina, which is an extract from the tonka bean.

The great therapeutical value of iodoform is due to its antiseptic and anæsthetic properties. The latter are not strongly marked, but it is certain that pain has been allayed by an application of iodoform in the form of powder, but whether this is due to the local anæsthesia, or to its great antiseptic properties by which it relieves the tissues of the irritating septic matter, is as yet an unsettled question. The antiseptic properties of iodoform are of the greatest importance, and the manner in which it acts is believed to be as follows (Hogyes and Niemeyer): "Iodoform, when applied to wounds, is first dissolved in the fats present in the tissues, from which free iodine is, according to the authors, gradually liberated." The action of iodoform is, therefore, due to the iodine, which in the nascent state has no irritating properties, such as are observed when iodine or its compounds are made use of. Behring (*Deutsch Med. Wochenschr, No.* 11) states that when iodoform is mixed with starch or flour, after fourteen days standing no free iodine is noticeable, and this even when mixed with acids or alkalies. Upon the application of heat, electricity, or substances which will readily effect oxidation, as peroxide of hydrogen, oil of turpentine, benzol, carbolic acid, etc., free iodine is liberated. The same is true when in contact with blood, but other tissues, such as connective tissue, the serum of blood, laudable pus, and nerve tissue, do not effect its decomposition. Fats in the fluid state dissolve iodoform and combine with it, whereby it is slightly altered, although no free iodine is liberated, but as decomposition sets in iodine is set free. In fresh and dry wounds, where no oxydizable matter is present, the iodóform remains unchanged, and the same is true when applied to unwounded granulations, but upon the exudation of blood free iodine develops. Iodoform, when in contact with blood out of the body acts upon the red blood corpuscles, making them scarlet red, while the iodoform assumes a bluish hue. When granules of starch are added they also become blue, but as the albumen of the blood acts upon the blood corpuscles, the blue stain is again removed from the starch. Binz (*Arch. f Path. Anat. and Physiol. Bd.* 48, *Heft* 3) has observed that iodoform in the form of powder, when applied to the messentary of the frog, reduces the emigration of colorless blood corpuscles, which is not the case when an oily solution of iodoform prepared in the dark has been made use of, and the prepared frog has also been kept in the dark for several hours. Thus iodoform vapor paralyzes and kills the white corpuscles. This action of iodoform in emigration can be accounted for by the other facts discovered by Binz, that iodoform, under the effect of daylight, is reduced in such a manner that iodine passes through the walls of the capillary blood-vessels without perceptibly affecting them, while it paralyzes and kills the white blood corpuscles which adhere to them. Vom Hoffer (*Wiener Medic. Wochenschr, No.* 38) regularly observed, after a hypodermic injection of iodoform in rabbits, a diminution of red corpuscles.

How iodine acts as an antiseptic has not been definitely settled, but as it belongs to the group of hyaloid bodies (chlorine, iodine, bromine and fluorine), which all have a very great affinity for hydrogen, it may be hypothetically explained that the free iodine combines with the hydrogen of the water present in every tissue; thus oxygen is set free, which in turn may neutralize septic matter.

Harmless as iodoform seems to be in certain quantities, yet cases of poisoning by the valuable drug have been recorded by Schede, Czerny, Langstein, Oberlander, and others, although the amount used was very large in comparison to that in which it is applied in dental practice. A short review of some of the cases may be of interest, even in this connection.

(TO BE CONTINUED.)

